# Ischemic stroke as the initial presentation of undiagnosed aortic coarctation in an adolescent: a case report

**DOI:** 10.1093/omcr/omag148

**Published:** 2026-08-03

**Authors:** Malegna Temesgen Garuma, Mihretie Jenber Tesfa, Leja Hamza Juhar, Tadele Zerga Chaka

**Affiliations:** Department of Internal Medicine, St. Paul’s Hospital Millennium Medical College, Swaziland Street, Gulele Sub-City, P.O. Box 1271, Addis Ababa, Ethiopia; Department of Internal Medicine, St. Paul’s Hospital Millennium Medical College, Swaziland Street, Gulele Sub-City, P.O. Box 1271, Addis Ababa, Ethiopia; Nephrology Unit, Department of Internal Medicine, St. Paul’s Hospital Millennium Medical College, Swaziland Street, Gulele Sub-City, P.O. Box 1271, Addis Ababa, Ethiopia; Department of Internal Medicine, St. Paul’s Hospital Millennium Medical College, Swaziland Street, Gulele Sub-City, P.O. Box 1271, Addis Ababa, Ethiopia

**Keywords:** coarctation of the aorta, bicuspid aortic valve, ischemic stroke, adolescent hypertension, congenital heart disease, case report

## Abstract

Coarctation of the aorta is a congenital cardiovascular anomaly that may remain undiagnosed until adolescence. Delayed diagnosis can result in serious complications, including systemic hypertension and cerebrovascular events. We report the case of a 16-year-old Ethiopian boy who presented with sudden-onset right-sided hemiparesis and expressive aphasia. Examination revealed a marked discrepancy between the upper and lower limb blood pressures and absent femoral pulses. Neuroimaging revealed a large left middle cerebral artery infarction. Transthoracic echocardiography and computed tomography angiography confirmed severe native coarctation of the aorta distal to the left subclavian artery, associated with a bicuspid aortic valve and extensive collateral circulation. The patient was treated for ischemic stroke and hypertensive urgency and was referred for definitive surgical correction. Ischemic stroke may be a rare initial manifestation of undiagnosed aortic coarctation in adolescents. Routine four-limb blood pressure measurements are essential for the early diagnosis and prevention of catastrophic outcomes.

## Introduction

Coarctation of the aorta (CoA) is a congenital narrowing of the thoracic aorta that accounts for approximately 5%–8% of congenital heart disease cases [1]. Although most cases of coarctation of the aorta (CoA) are identified in infancy, some remain undiagnosed until adolescence or adulthood. Early detection relies on careful physical examination, particularly the assessment of the radio-femoral delay and differences in upper- and lower-limb blood pressure [2]. Transthoracic echocardiography serves as an initial screening tool, but definitive anatomical assessment requires CTA or MRA [3]. Classic findings, such as blood pressure discrepancies between limbs and absent femoral pulses, are often missed during routine practice, contributing to delayed diagnosis.

Patients with mild or discrete coarctation may remain asymptomatic for years because extensive collateral circulation and compensated hemodynamics preserve distal perfusion despite significant anatomical obstruction. Consequently, diagnosis may be delayed until adolescence or adulthood when hypertension, exercise intolerance, or complications develop. Delayed diagnosis is clinically significant because prolonged obstruction causes persistent systemic hypertension, leading to cardiovascular complications [1]. Beyond systemic effects, coarctation carries important neurological risks. Chronic hypertension and altered cerebral hemodynamics associated with CoA increase the risk of ischemic stroke, intracranial aneurysm formation, and hemorrhagic stroke. Patients with CoA may develop cerebrovascular abnormalities and altered intracranial vascular characteristics, further contributing to stroke risk. Extensive collateral circulation may partially compensate for distal hypoperfusion and mask symptoms, further delaying diagnosis [4–6]. This case highlights the consequences of a delayed diagnosis of native aortic coarctation and underscores the importance of routine blood pressure and pulse examinations in young patients, as well as heightened clinical vigilance for potential cerebrovascular complications.

## Case report

A 16-year-old male patient from Ethiopia presented with a two-week history of sudden-onset right-sided body weakness that was maximal at onset while he was sitting. This was accompanied by difficulty in speaking and right-sided facial droop consistent with upper motor neuron facial weakness. The patient had a history of intermittent headaches since childhood, which became severe and persistent one week prior to the onset of weakness. He had been experiencing palpitations for the past five years.

Vital signs showed a blood pressure (BP) of 150/100 mmHg in the upper limbs, whereas the lower limb BP was 80/50 mmHg. The pulse rate was 90 beats per minute and regular. The femoral and distal lower limb pulses were absent. Cardiac examination revealed an ejection systolic murmur in the aortic area and a systolic murmur over the left infraclavicular region. Neurological examination revealed a Glasgow Coma Scale score of 11/15 (E4V1M6) with expressive aphasia, right-sided weakness (0/5 in the upper and 1/5 in the lower extremity), and hypertonia with an upgoing plantar reflex, while left-sided power was normal (5/5).

The complete blood count and routine serum chemistry results were normal. Chest radiography revealed rib notching and an increased cardiothoracic ratio (Fig. 1). Laboratory investigations were unremarkable. Electrocardiography showed left ventricular hypertrophy. Prolonged rhythm monitoring was not done. Transthoracic echocardiography revealed a bicuspid aortic valve with moderate stenosis, concentric left ventricular hypertrophy, and Doppler evidence of aortic coarctation. No intracardiac thrombus, shunts, or valvular vegetations were identified. CT angiography of the thoracic aorta confirmed severe discrete narrowing of the descending aorta distal to the left subclavian artery with extensive collateral vessels (Fig. 2). Brain CT and magnetic resonance imaging revealed a large left middle cerebral artery territory infarction with mass effects (Fig. 3). CT angiography/MRA of intracranial vessels was not performed.

**Figure 1:**
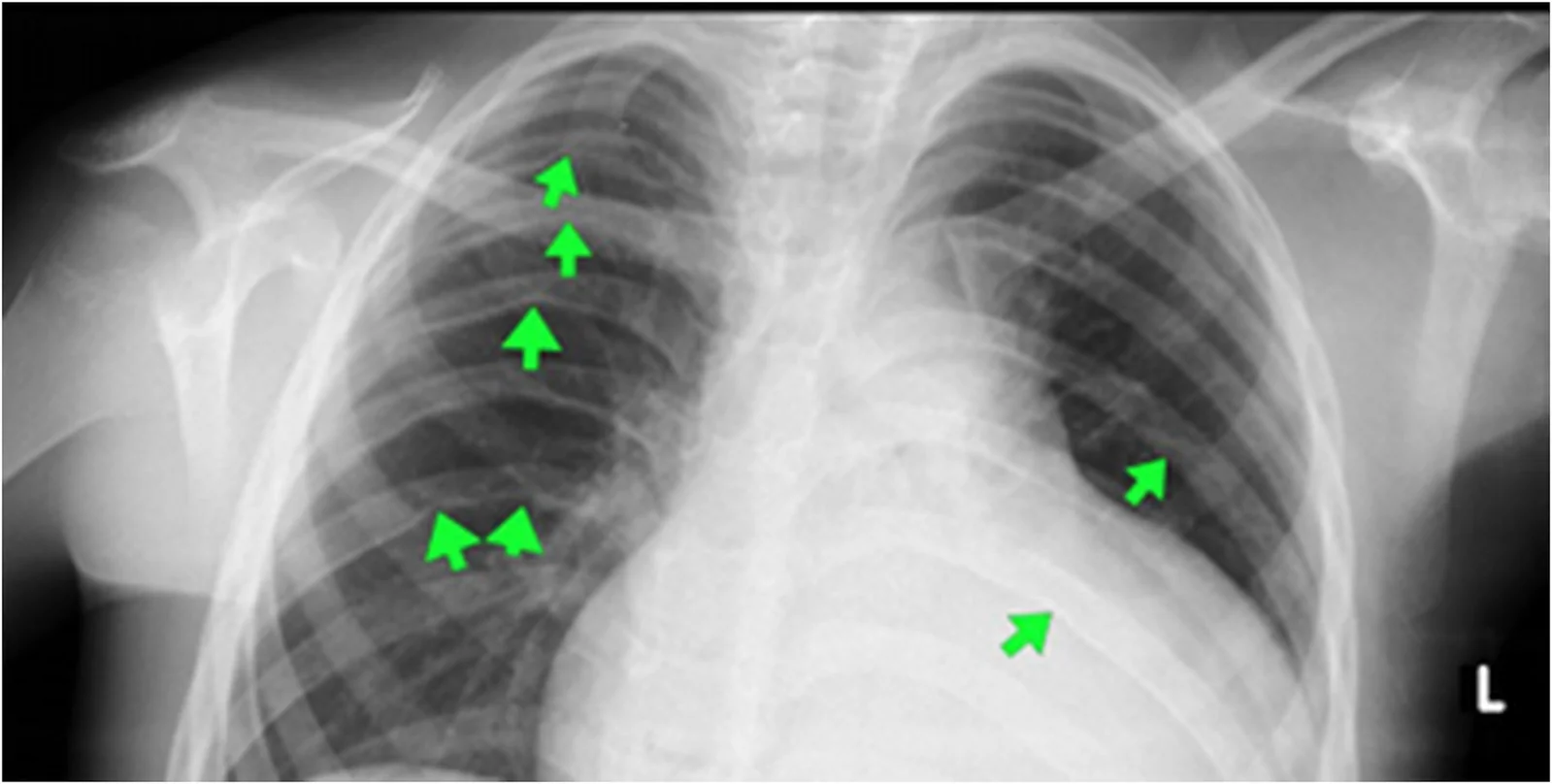
Chest X-ray demonstrating right-sided rib notching (fourth to seventh ribs, indicated by arrows) and an increased cardiothoracic ratio, consistent with chronic cardiac adaptation to coarctation of the aorta.

**Figure 2:**
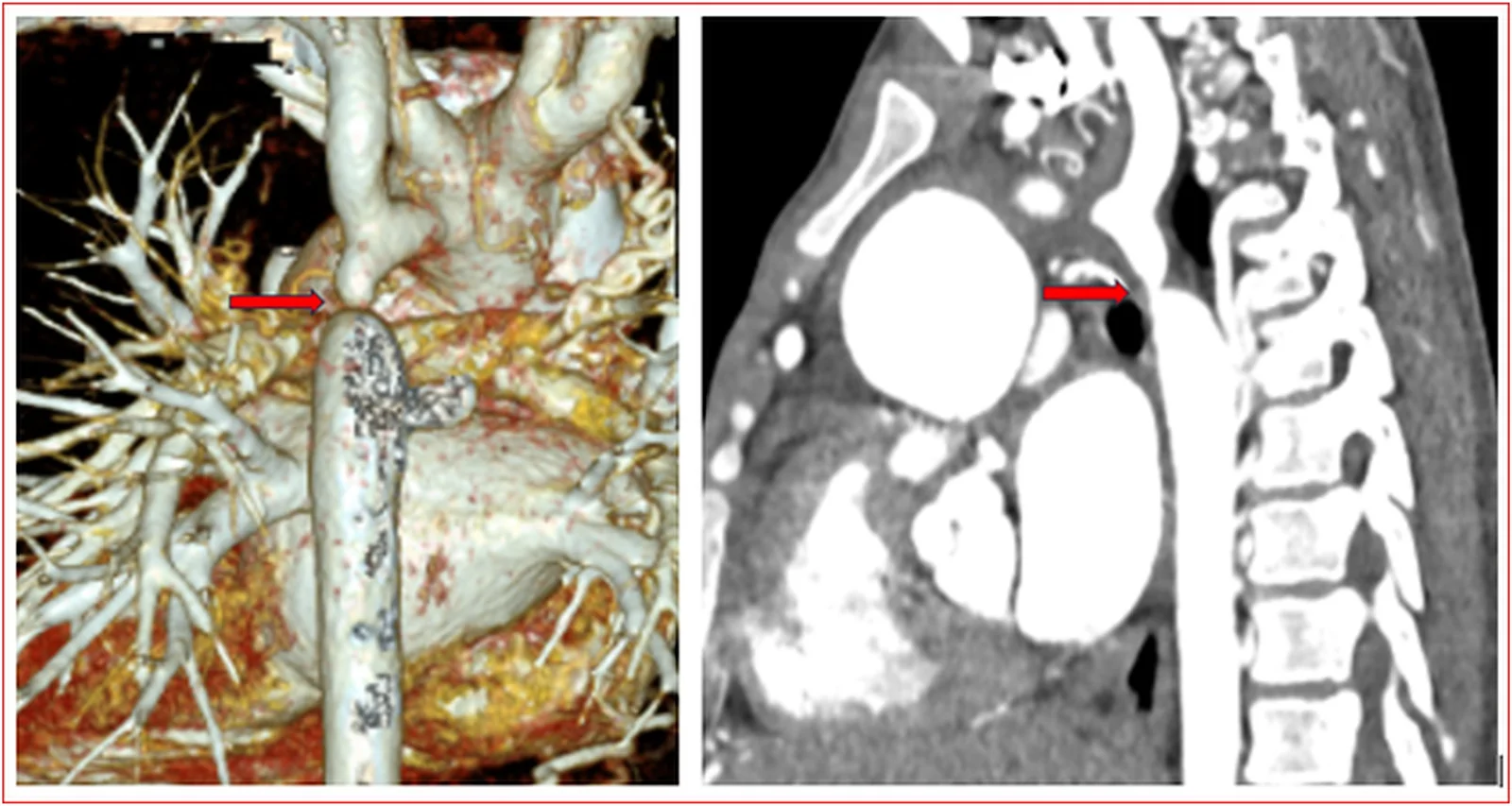
Computed tomography angiography of the thoracic aorta revealing discrete severe narrowing of the descending aorta distal to the left subclavian artery (indicated by red arrows), with extensive collateral vessels visible around the site of narrowing.

**Figure 3:**
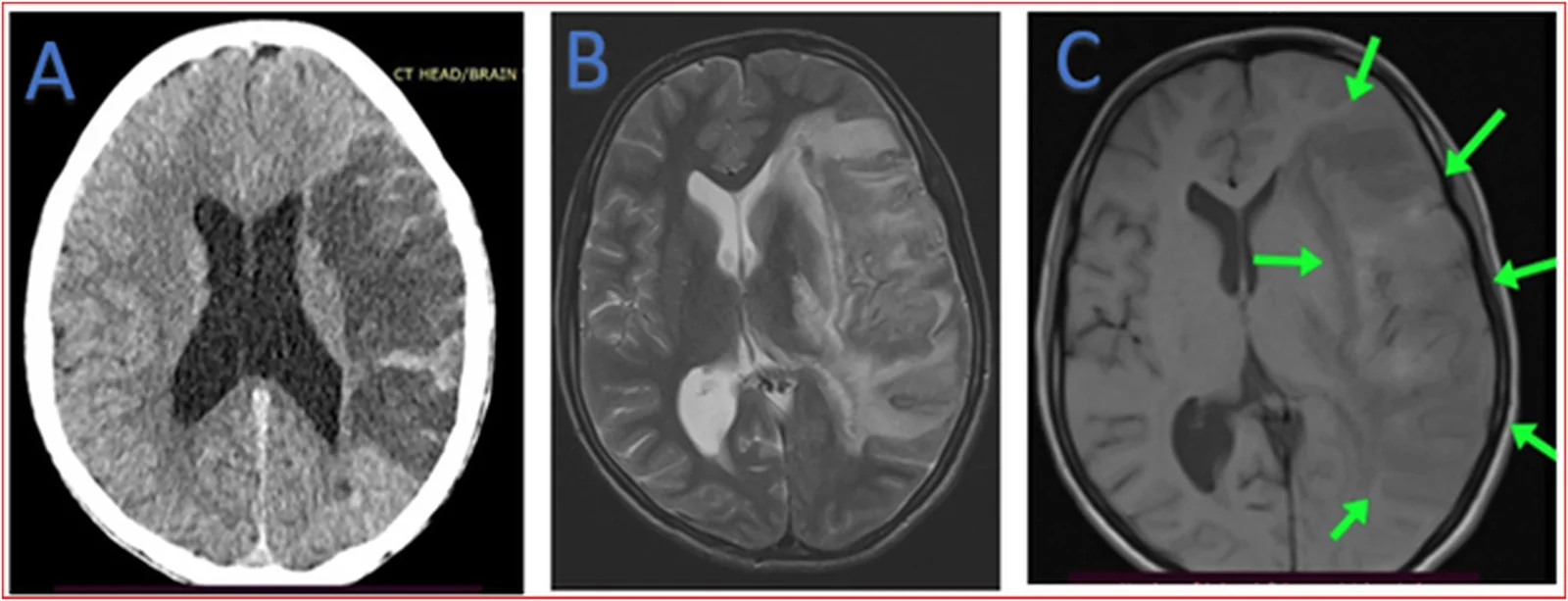
(A) Non-contrast brain computed tomography scan demonstrating a large subacute infarction in the left middle cerebral artery (MCA) territory, with hypodensity involving the frontoparietal region, associated with mass effect, midline shift, effacement of the ipsilateral lateral ventricle, and marginal petechial hemorrhage. (B) Axial T2-weighted brain magnetic resonance imaging showing extensive hyperintensity in the left MCA territory involving the frontoparietal cortex, consistent with ischemic infarction. (C) Axial T1-weighted brain magnetic resonance imaging demonstrating corresponding hypointense changes in the left MCA territory with mass effect; the extent of cortical and subcortical involvement is indicated by the area of signal change.

The patient was treated for subacute ischemic stroke and hypertensive urgency with supportive care and antihypertensive therapy. Following stabilization, the patient was referred for definitive surgical correction of the aortic coarctation. At the time of referral, the patient had partial neurological improvement and controlled blood pressure.

## Discussion

This case report describes a late presentation of native coarctation of the aorta (CoA) in an adolescent male complicated by ischemic stroke, illustrating the potentially serious consequences of delayed diagnosis.

The coexistence of a bicuspid aortic valve (BAV) may further increase cardiovascular risk because of associated aortopathy and vascular dysfunction [4]. Although rare as an initial clinical presentation, coarctation of the aorta is associated with increased risk of premature ischemic stroke in children and young adults. In a national population-based study, adults with coarctation of the aorta experienced stroke at a mean age approximately 16 years younger than the general population [7]. This underscores the importance of screening for secondary causes of adolescent hypertension.

While hypertension is a significant contributing factor in this case, the etiology of stroke in coarctation is complex and multifactorial. The large left middle cerebral artery territory infarction observed in this patient is atypical for purely hypertensive stroke, suggesting additional mechanisms. In patients with CoA, elevated blood pressure results from fixed mechanical obstruction, altered baroreceptor sensitivity, neurohormonal activation, and proximal vascular dysfunction [4, 8]. However, other mechanisms may contribute to stroke risk in this population, including: (I) altered cerebral hemodynamics secondary to proximal hypertension and distal hypoperfusion, (II) cerebrovascular abnormalities and arterial remodeling associated with chronic hypertension, (III) cardioembolism, particularly in the presence of undetected arrhythmias or other occult embolic sources, and (IV) unrecognized thrombophilic disorders that were not evaluated in this case. Although transthoracic echocardiography identified no intracardiac thrombus or vegetation, absence of intracranial vascular imaging and comprehensive thrombophilia workup limits definitive determination of stroke etiology in this case. Prolonged exposure to elevated blood pressure does predispose patients to left ventricular hypertrophy, premature vascular disease, and cerebrovascular events, but these should be understood as part of a broader pathophysiological process rather than the sole mechanism of stroke [1, 5, 7].

Definitive management of CoA includes surgical repair or transcatheter intervention, which are associated with improved long term outcomes [9]. However, patients diagnosed later in life are more likely to have persistent hypertension even after successful correction, reflecting irreversible vascular remodeling [10]. The patient was referred for definitive correction of the coarctation after stabilization of the acute neurological condition and appropriate blood pressure control was achieved.

Coarctation of the aorta remains a potentially overlooked diagnosis in adolescents and young adults despite the availability of straightforward diagnostic techniques. This case illustrates how careful physical examination, including four-limb blood pressure measurement and femoral pulse assessment, can identify previously undiagnosed coarctation of the aorta presenting with a serious cerebrovascular complication. Early recognition of secondary causes of hypertension, including congenital heart disease, is essential for preventing catastrophic complications.

### Learning points

- Adolescents with hypertension or unexplained neurological deficits should be evaluated for secondary causes, including coarctation of the aorta.
- Four-limb blood pressure measurement and femoral pulse assessment are essential for early diagnosis.
- Delayed diagnosis of coarctation of the aorta, particularly with a bicuspid aortic valve, may contribute to cerebrovascular complications through hypertension, altered cerebral hemodynamics, and vascular remodeling.
